# p-n Control of AlMgB_14_-Based Thermoelectric Materials by Metal Site Occupancy

**DOI:** 10.3390/ma12040632

**Published:** 2019-02-20

**Authors:** Takuya Fujima, Natsuki Shimizu, Hideki Arimatsu

**Affiliations:** Department of Mechanical Engineering, Tokyo City University, Tokyo 158-8557, Japan; g1681110@tcu.ac.jp (N.S.); hideari.861@gmail.com (H.A.)

**Keywords:** AlMgB_14_, higher boride, thermoelectric properties, Rietveld refinement, first-principle calculation

## Abstract

The mechanism for the p-n control of AlMgB_14_-based thermoelectric material was investigated using Rietveld refinement and the first principle calculation. The p- and n-type AlMgB_14_-based thermoelectric materials were prepared by spark plasma sintering (SPS) with changing raw powder mixture ratio. Temperature dependence of Seebeck coefficient and electrical conductivity were different between the two types of samples. Seebeck coefficient shifted from positive to negative with increasing the number of valence electrons in the metal sites calculated by the metal site occupancy. The density of states and electron density distribution indicated that the electrons transfer from metal atoms to the B atoms.

## 1. Introduction

Since the exhaustion of fossil fuels has been discussed as a serious problem for a long time, it is incontrovertibly necessary to reduce energy loss and improve the usage efficiency of the fuels [1,2,3,4,5]. The thermoelectric generation that can convert heat into electricity is gathering much attention as a promising candidate for a solution concerning the energy issue [6]. The performance of the thermoelectric materials is evaluated by the dimensionless figure of merit *ZT* = *S*^2^*σT*/*κ*, where *S* is the Seebeck coefficient, *σ* is the electrical conductivity, and *T* and *κ* are the temperature and the thermal conductivity, respectively [7]. Therefore, the large *S*, high *σ*, and low *κ* are advantageous for large *ZT*. A practical thermoelectric generation module consists of both p-type and n-type thermoelectric materials [8].

Boron-rich materials consisting of the B_12_ icosahedral cluster are known to exhibit semi conductive properties, low heat conductivity, and high temperature stability [9,10,11,12,13,14]. Furthermore, it has been noticed that the decrease in the absolute value of the Seebeck coefficient in accordance with the increasing temperature is minimal [13,15,16,17,18,19]. This is attributed to the constant number of carriers in accordance with the temperature increase; the mechanism of electric conduction involves variable range hopping due to carrier jumping between clusters while mobile [20]. Furthermore, in the variable range hopping conduction, the frequency of the carrier jumps increases in accordance with the increasing temperature. Consequently, the electrical conductivity, as in other semiconductors, increases at higher temperatures. As described previously, it is known that regular B_12_ icosahedral cluster solids have comparatively high thermoelectric properties at a high temperature, making them promising high-temperature thermoelectric materials. However, most B_12_ icosahedral cluster solids have comparatively high thermoelectric properties at a high temperature, making them promising high-temperature thermoelectric materials. However, most B_12_ icosahedral cluster solids are p-type materials and the development of an n-type material to create a pair is desirable [16,21,22,23,24].

An example of the B_12_ icosahedral cluster solid is AlMgB_14_, of which a unit cell includes four Al sites, four Mg sites, and 56 B sites. Similar to other B_12_ icosahedral cluster solids, AlMgB_14_ is expected to function as a thermoelectric conversion material at high temperatures due to the small decrease in the Seebeck coefficient in accordance with the increasing temperature and the material’s high chemical stability at high temperatures. Moreover, it has been reported that the Seebeck coefficient of AlMgB_14_ is 300–400 μV/K, a high positive value compared to multiple thermoelectric conversion materials [9,25]. In recent years, we developed an AlMgB_14_-based thermoelectric material exhibiting n-type characteristics by nonstoichiometric composition [26,27]. However, we have not clarified the detailed reason for our n-type AlMgB_14_-based materials. Therefore, herein, to elucidate the mechanism of an n-type property for AlMgB_14_-based thermoelectric conversion materials, the dependence of the thermoelectric properties on the site occupancy ratio within the crystal was investigated.

## 2. Materials and Methods

In this work, all samples were prepared using the powder metallurgy method. The following raw materials were used: Al (99.9% purity, Kojundo Chemical Laboratory Co., Ltd., Saitama, Japan), Mg (99.5% purity, Kojundo Chemical Laboratory Co., Ltd., Saitama, Japan), and amorphous B (96.2% purity, H.C. Starck Ltd., Munich, Germany). These powders were weighed at a mixing ratio listed in Table 1. The weighed raw material powders were mixed using a V-shape mixer at 20 rpm for 30 min. Thereafter, the mixed powder was sintered using a Spark Plasma Sintering (SPS) apparatus (Fuji Electronic Industrial Co., Ltd., SPS-515S, Osaka, Japan) under a compacting pressure of 30 MPa. A graphite die and punch were used for the sintering in an Ar atmosphere at a holding temperature of 1773 K for 25 min. The sintered body was subjected to heat treatment at 973 K for 3 h. The heat-treated sintered body was used as a sample in this research.

Powder X-ray diffraction (XRD) analysis was performed on each of the samples. The powders were pulverized by fine grinding in a planetary ball mill (Ito Seisakusho Co., Ltd., LA-P04, Tokyo, Japan), with balls and a pot comprising SUS440C and SUS304, respectively, at 200 rpm for 6 h. SUS304 and SUS440C mixed during the pulverization were removed using hydrochloric acid. For powder X-ray diffraction, measurements were performed with a Cu Kα-ray source using a diffraction angle range of 10° < 2θ < 120° in conjunction with an X-ray diffractometer (Bruker AXS Ltd., New D8 ADVANCE, Massachusetts, USA). The obtained pattern was used to define the crystal structure via Rietveld analysis using RIETAN-FP [28]. To measure the Seebeck coefficient and electrical conductivity, a thermoelectric characteristic evaluation device (Advance-RIKO, Inc., ZEM-1, Kanagawa, Japan) was used.

In addition, first-principle band calculations were performed based on the density functional theory using local spin density approximation (LSDA) with the augmented plane wave (APW) + lo method by using WIEN 2k [29]. The unit cell was doubled in the a-axis and c-axis directions, and calculations were performed for a 2 × 1 × 2 supercell.

## 3. Results and Discussion

### 3.1. Rietveld Analysis

**Figure 1:**
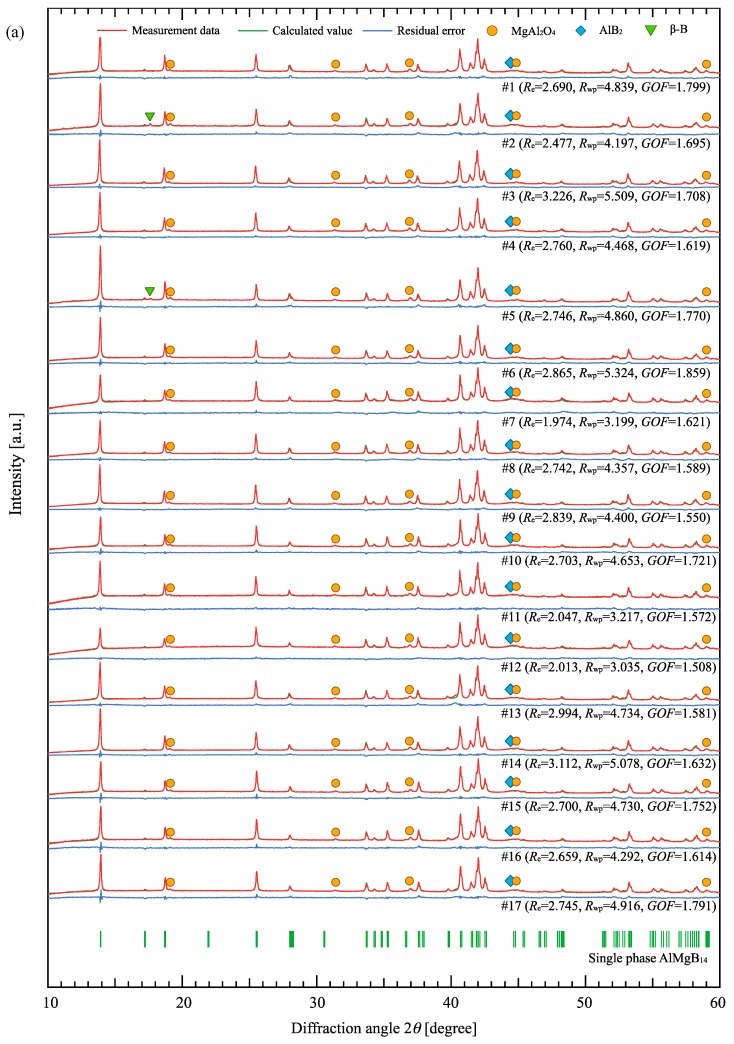

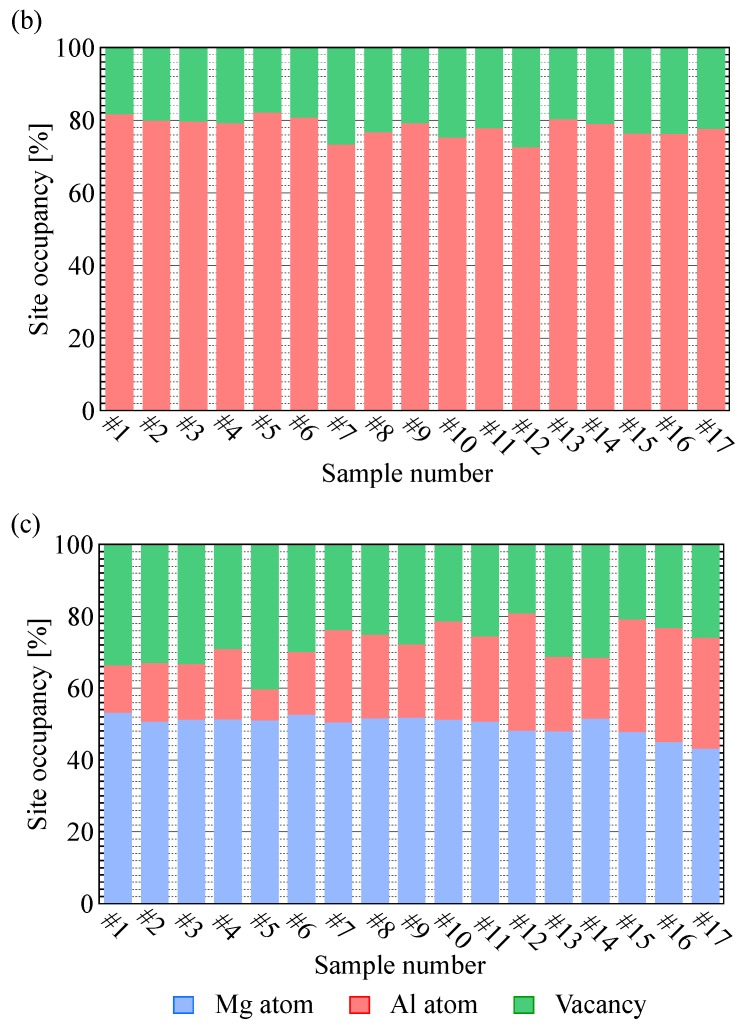
(**a**) X-ray diffraction (XRD) patterns of all the samples with calculated pattern and residual by Rietveld refinement and the calculated metal site occupancy in (**b**) Al site and (**c**) Mg site. The indexes beside each XRD pattern are the R factors in the refinement.

Figure 1a shows the XRD patterns for all the samples. The main peaks therein were attributed to AlMgB_14_ and some to a small amount of AlB_2_ and β–B. The calculated patterns by Rietveld refinement well matched to the experimental ones with nearly flat residual patterns as shown in the figure. 

The Al and Mg site occupancies calculated as per Rietveld refinement are shown in Figure 1b,c, respectively. As shown in the figures, both sites remain approximately 20–40% vacancy, and the Mg site is occupied by both Mg and Al other than the Al site that only contains Al atoms. On comparing the Al site occupancy and the Mg atom occupancy in the Mg site, considerable differences between the samples were observed, suggesting a large contribution of the mixing ratio of the raw material powder to the Al atom occupancy ratio in the Mg site.

### 3.2. Thermoelectric Properties

**Figure 2:**
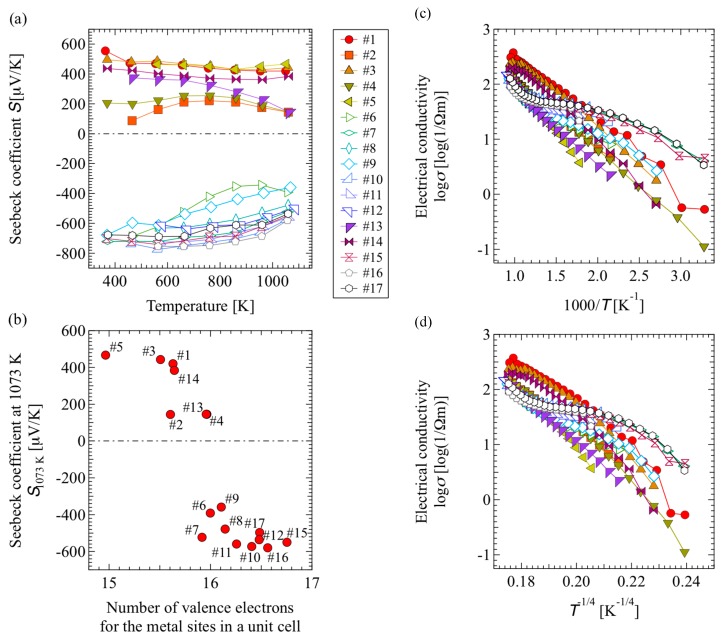
Seebeck coefficient as a function of (**a**) the measurement temperature and (**b**) number of valence electrons for the metal sites in a unit cell. In (**a**), the filled plots indicate p-type samples and the open plots indicate n-type ones. Temperature dependence of electrical conductivity is plotted against (**c**) T^−1^ and (**d**) T^−1/4^ in accordance with Mott’s law of variable range hopping.

Figure 2 shows (a) the temperature dependence and (b) the number of valence electrons (NVE) in the metal sites dependence of the Seebeck coefficient. The NVE in the metal sites was calculated by considering NVE of the Al and Mg atoms as 3 and 2, respectively, based on the metal site occupancy shown in Figure 1. 

Some of the p-type samples, #2, #4, and #13, exhibited a temperature dependence of the Seebeck coefficients that is similar to that of undoped β-boron, whereas the others, #1, #3, #5, and #14, did that of metal-doped β-boron. Both the impurity-free and metal-doped β-boron reportedly have a mechanism of electric conduction wherein band conduction and variable range hopping conduction contribute [30]. In contrast, the Seebeck coefficient of all n-type materials exhibited a tendency to decrease the absolute value in accordance with the increasing temperature. This tendency is similar to that of non-degenerate semiconductors where band conduction is predominant.

Figure 2b indicates a transition from p-type to n-type, accompanying the increase in the NVE in the metal site. Furthermore, the boundary between the p-type and n-type is at approximately 16 for the NVE in the metal site such that samples with lower numbers exhibit p-type characteristics, whereas those with higher numbers exhibit n-type characteristics.

Figure 2c,d show the temperature dependence of the electrical conductivity of the samples. Figure 2c shows the relation between log σ and 1000/T, whereas Figure 2d shows the relation between log σ and T^−1/4^. In Figure 2c, the temperature dependence of the electrical conductivity of all p-type samples (filled markers) shows a tendency close to linearity at 1000/T > 1.2; however, in the high-temperature region of 1000/T < 1.2, curvilinear plots were obtained. In contrast, in Figure 2d, the temperature dependence of the electrical conductivity of all p-type samples was curvilinear over the entire temperature range. Since the plots in Figure 2c,d show a curvilinear tendency, the electrical conductivity of the p-type samples cannot be considered to be solely derived from either band conduction or variable range hopping conduction. Based on the temperature dependence of the aforementioned Seebeck coefficient and the electrical conductivity, it can be considered that band conduction and variable range hopping conduction act in accordance with the electrical conduction mechanism of p-type samples. 

With regard to the electrical conductivity of n-type materials, according to Figure 2c,d, a low temperature dependence was observed in the intermediate temperature ranges of 1.2 < 1000/T < 2.2 and 0.19 < T^−1/4^ < 0.22, respectively. This tendency is similar to the temperature dependence of electrical conductivity in non-degenerate impurity semiconductors. Furthermore, the temperature dependence of the Seebeck coefficient in n-type samples was similar to that in non-degenerate semiconductors. From the temperature dependence of the Seebeck coefficient and the electrical conductivity, we infer that electrical conduction in an n-type sample follows an electrical conduction mechanism similar to that observed in non-degenerate impurity semiconductors.

### 3.3. Results of First-Principle Band Calculation

**Figure 3:**
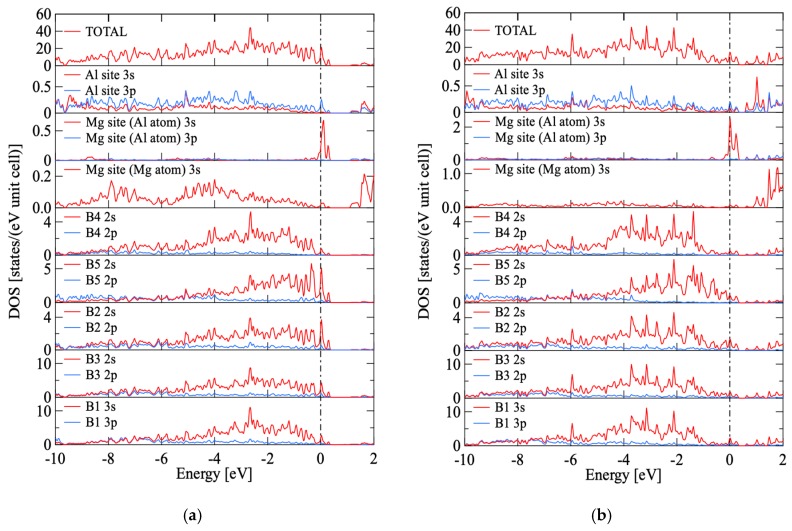
TDOS and site-projected PDOS for Al atom in Al site, Mg atom in Mg site, Al atom in Mg site, B4 site, B5 site, B2 site, B3 site, and B1 site of (**a**) #5 and (**b**) #15. The Fermi energy is at the zero of energy.

Herein, first-principle band calculations were performed using the Rietveld analysis results of sample #5 (showing p-type characteristics) and sample #15 (n-type) as representative examples. We used the detailed parameters for the calculation listed in Table 2. Figure 3a,b show the total density of states (TDOS) computed as per the first-principle band calculation and the projected density of states (PDOS) for each atom at each site for sample #5 and #15. The Fermi level *E*_F_ was set to 0 eV. 

The PDOS spectral shapes of all B sites and Al site are similar to each other for both of the p and s orbitals. This indicates that hybrid orbitals are formed over the Al and B sites. Furthermore, the similarity between TODS and PDOS at the Al and B sites suggests the hybrid orbital predominant in this material.

**Figure 4:**
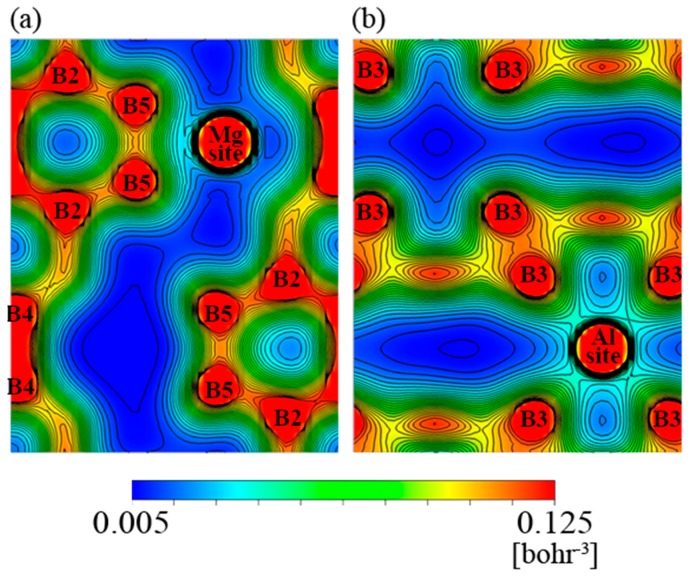
Electron density distribution in the planes containing (**a**) Mg sites and (**b**) Al sites of #5.

**Figure 5:**
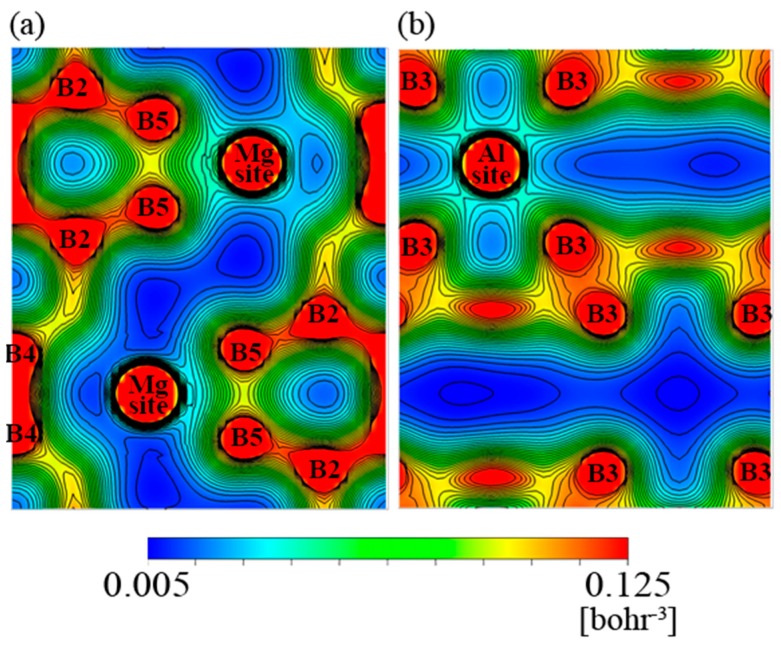
Electron density distribution in the planes containing (**a**) Mg sites and (**b**) Al sites of #15.

Figure 4 and Figure 5 demonstrate the electron density distribution of sample #5 and #15, respectively. The (a) piece in both figures show a cross-sectional crystal plane containing a Mg site, and (b) ones show a crystal plane containing an Al site. According to the figures, the electron density between the metal and boron atoms is distributed in the spherical symmetry, except for the contact portion with adjacent atoms. It is therefore clear that the bond between the metal and boron atoms is ionic. Between the boron atoms, the electron density has a strong directionality, suggesting that the bond between the boron atoms is covalent. 

Based on a comparison between Figure 4 and Figure 5, it can be inferred that the electron density in the B_12_ icosahedral cluster of the n-type (sample #15) is totally higher than that of the p-type (sample #5) because the electron-poor area colored by deep blue in the figures is smaller for the former. Since Figure 2b shows that the number of valence electrons in the metal sites is larger in the n-type sample than in the p-type sample, we consider that the electrons transferred from the metal atoms to the boron atoms. 

On the other hand, the electron density around the B5 site which is not included in the B_12_ icosahedral cluster and that around the B2 site which is in the cluster and bound to the B5 site appeared to be larger for #5 than for #15. This is consistent with a result about YAlB_14_, a material that has the same crystalline structure as AlMgB_14_ [31], where the Al-site occupancy reportedly coincides with the electron density around B5 and B2 sites, since Figure 1 shows that the Al site occupancy of sample #5 is larger than that of #15. This also indicates that electrons can transfer from the metal site to the boron sites in the AlMgB_14_ crystal.

Furthermore, the AlMgB_14_-based material changes its thermoelectric type between n and p around a number of valence electron in a unit lattice of 16 as shown in Figure 2b. The number is identical to that of electron deficiencies in B atoms per an AlMgB_14_ unit cell [32]. Since the results of first-principle calculations suggest electron supply from the metal sites to the boron sites, it can be assumed that manifestation of n-type characteristics is due to a reduction in the electron deficiency of the B bond.

In comparison with our previous work [27], we kept samples 2.5 times longer at the sintering temperature and carried out further annealing process to make samples more of equilibrium in the preparation process. The n-p type transition occurred around an NVE in the metallic sites of ca. 15 in the previous work other than this work (ca. 16). This indicates the boronic framework in the AlMgB_14_-based materials changes its electron deficiencies during its synthesis process that is possibly due to the impurity and defect elimination from the framework. This work reached a theoretical consistency about the mechanism of n-p transition of the AlMgB_14_-based materials.

The above discussion is based on the metal site occupancy obtained sole by the Rietveld refinement. It could be better if a more precise composition analysis method can be used together. Such a method, like inductively coupled plasma mass spectrometry (ICPMS), nuclear magnetic resonance (NMR), and Rutherford back scattering (RBS), can be used as composition analysis only if the AlMgB_14_ phase can be selectively measured, but multiphase and polycrystalline materials in this study are not suitable because the effect of the secondary phase and grain boundaries cannot be eliminated. Therefore, the XRD and Rietveld refinement is the only practical method applicable in this study, and the dependence of thermoelectric n-p type on the number of valence electron based on the occupancy rate is consistent with theory. This indicates that the analysis has been reasonable.

## 4. Conclusions

Herein, we studied the mechanism of the n-p control for AlMgB_14_-based thermoelectric materials. The synthesized compounds exhibited a transition between p-type and n-type thermoelectric properties in accordance with the number of valence electrons that was calculated from metal-site occupancy obtained by Rietveld analysis. The experimentally revealed transition point was consistent with the number of electron deficiencies through B atoms, that indicates electron transfer from the metal sites to B sites in the AlMgB_14_-based materials. This electron transfer was also supported by the first-principle band calculations that indicated the valence electrons transfer from the metal atoms to the boron atoms. 

## Figures and Tables

**Table 1:** Raw material mixture ration.

Sample	Raw Material Mixture Ration [Molar Ration]				
Number	Al	:	Mg	:	B
#1	0.98	:	1.06	:	14
#2	1.00	:	0.99	:	14
#3	1.01	:	1.02	:	14
#4	1.01	:	1.11	:	14
#5	1.02	:	0.92	:	14
#6	1.02	:	0.96	:	14
#7	1.02	:	1.01	:	14
#8	1.02	:	1.06	:	14
#9	1.04	:	1.04	:	14
#10	1.04	:	1.12	:	14
#11	1.04	:	1.16	:	14
#12	1.10	:	1.06	:	14
#13	1.12	:	0.99	:	14
#14	1.12	:	1.11	:	14
#15	1.14	:	1.06	:	14
#16	1.18	:	1.06	:	14
#17	1.22	:	1.06	:	14

**Table 2:** Lattice constant and crystal structure parameter for the first principle calculation based on (**a**) sample #5 and (**b**) #15.

(**a**)							
#5	Lattice Constant [Å]	a	5.85063				
		b	10.32511				
		c	8.11847				
	Crystal Structure Parameter	Site	Fractional Coordinate			Atom	Occupancy [%]
			x	y	z		
		B1	0.15985	0.56183	0.16043	B	100.00
		B2	0	0.08655	0.17090	B	100.00
		B3	0.25153	0.07959	0.04495	B	100.00
		B4	0	0.66767	0.01982	B	100.00
		B5	0	0.15787	0.37898	B	100.00
		Al	1/4	1/4	1/4	Al	82.12
		Mg	0	1/4	0.64651	Mg	51.07
						Al	8.54
(**b**)							
#15	Lattice Constant [Å]	a	5.85399				
		b	10.30819				
		c	8.11090				
	Crystal Structure Parameter	Site	Fractional Coordinate			Atom	Occupancy [%]
			x	y	z		
		B1	0.16209	0.56242	0.16089	B	100.00
		B2	0	0.08605	0.17424	B	100.00
		B3	0.24768	0.07860	0.04583	B	100.00
		B4	0	0.66614	0.01407	B	100.00
		B5	0	0.15344	0.38171	B	100.00
		Al	1/4	1/4	1/4	Al	76.37
		Mg	0	1/4	0.63967	Mg	47.77
						Al	31.39
